# Predictors of sedentary behaviour in adults: the role of social media addiction and physical activity – a cross-sectional study from Turkey

**DOI:** 10.7189/jogh.16.04206

**Published:** 2026-07-10

**Authors:** Sema Büğüşan Oruç, Nuran Akdağ Mirzaoğlu, Zeynep Yıldırım, Kamile Uzun Akkaya, Bülent Elbasan, Yavuz Yakut

**Affiliations:** 1 Department of Physiotherapy and Rehabilitation, Iğdır University, Iğdır, Turkey; 2 Department of Physiotherapy and Rehabilitation, Gazi University, Ankara, Turkey; 3 Department of Therapy and Rehabilitation, Iğdır University, Iğdır, Turkey; 4 Department of Nursing, Iğdır University, Iğdır, Turkey; 5 Department of Physiotherapy and Rehabilitation, Hasan Kalyoncu University, Gaziantep, Turkey

## Abstract

**Background:**

Sedentary behaviour is increasingly recognised as a major global public health concern due to its well-established associations with adverse health outcomes. Despite the rapid rise in digital media use, research examining the relationship between digital media use and sedentary behaviour in adult populations remains limited, especially in developing countries such as Turkey, where digital access and usage patterns are rapidly evolving. Therefore, to examine the associations between social media addiction and sedentary behaviour and to identify the predictors of sedentary behaviour in adults.

**Methods:**

This cross-sectional analytical study was conducted online with volunteer participants between April and June 2025. A total of 442 individuals aged 18–65 years living in Turkey participated. Data were collected using the sociodemographic form, the Bergen Social Media Addiction Scale, the Sedentary Behavior Scale, and the International Physical Activity Questionnaire-Short Form.

**Results:**

Social media addiction was weakly positively correlated with sedentary behaviour (r = 0.294, *P* < 0.001) and negatively correlated with physical activity (r = −0.124, *P* = 0.009), while no significant correlation was found between sedentary behaviour and physical activity (r = 0.020, *P* = 0.679). In a four-step hierarchical regression analysis, demographic variables explained 9.5% of the variance in sedentary behaviour. Physical activity was not a significant contributor (ΔR^2^ = 0.007, *P* > 0.05), whereas daily internet use time (ΔR^2^ = 0.109, *P* < 0.001) and social media addiction (ΔR^2^ = 0.007, *P* < 0.05) significantly improved the model.

**Conclusions:**

Hierarchical regression analyses showed that daily internet use was the strongest predictor of sedentary behaviour, while social media addiction had a small but significant effect. Physical activity was not a significant predictor, and several sociodemographic factors (age, gender, education, income, and employment) were also associated with sedentary behaviour. Overall, sedentary behaviour appears to be influenced by both demographic and behavioural factors, with digital addiction contributing modestly beyond traditional predictors.

Physical activity has positive effects on human health, such as protecting cardiovascular health, extending lifespan, and reducing cancer risk [1]. Individuals who have become physically inactive are at risk of facing health problems [2]. Increasing physical activity levels alone is not enough to protect against these risks. Indeed, the concept of ‘active couch potato’ has been defined in the literature. According to this definition, individuals with high levels of physical activity may have long-term sedentary behaviour. This distinction highlights the need to evaluate physical activity and sedentary behaviour simultaneously rather than considering them as opposite ends of the same continuum [3,4]. Sedentary behaviour refers to waking activities characterised by low energy expenditure (≤1.5 metabolic equivalent units) performed while sitting, reclining, or lying [5]. Evidence indicates that sedentary behaviour is associated with adverse health outcomes independently of physical inactivity. Higher levels of sedentary behaviour have been linked to increased risks of all-cause mortality, chronic kidney disease, cardiovascular disease, and cancer [6,7].

In response to growing concerns about movement-related health risks, public health strategies have evolved from focusing solely on physical activity to adopting a broader ‘24-hour movement behaviour’ framework that integrates physical activity, sedentary behaviour, and sleep within a full-day perspective [8–10]. Within this framework, all behaviours that may influence movement patterns throughout the day become important. In particular, excessive use of technological devices may exacerbate sedentary behaviour throughout the day and disrupt the overall balance of daily movement behaviours [11]. Indeed, an estimated 67.9% of the global population uses the internet [12], and internet use is even more prevalent in Turkey. According to the Turkish Statistical Institute, 90.9% of adults aged 16–74 in Turkey report using the internet [13] and spend approximately three hours on social media platforms [14]. However, the amount of time spent on social media alone is not sufficient to define addiction. A broader conceptual perspective digital addiction is generally defined as the persistent and compulsive use of digital technologies that significantly interferes with daily functioning.[15]. This umbrella term encompasses specific subtypes such as internet addiction, smartphone addiction, gaming addiction, and social media addiction [16–19]. Among these, social media addiction often manifests as a compulsive urge to repeatedly check or update online profiles, even when social media use undermines personal or professional responsibilities [20]. The World Health Organization has formally recognised technology addiction as a global problem, stating that addictive internet use leads to a loss of the ability to manage and balance time, energy, and attention [20].

Digital addiction is commonly studied in children, adolescents, and young adults [10,21–24]. In these age groups, screen time has negatively affected the developing brain; special attention has been given to this age group [22]. Furthermore, adolescents are more susceptible to digital addiction because their self-control and psychological maturity are not fully developed [10,25]. However, there is a growing need to investigate digital addiction in adults [26,27]. Social, psychological, and environmental factors can play a role in the emergence of addiction [22]. Adults frequently experience heightened levels of stress and anxiety and may increase screen use as a coping mechanism to escape these pressures [22,28]. Additionally, increasing digitalisation among older adults and their pursuit of social interaction through social media platforms further contribute to this phenomenon [11]. In addition, identifying digital addictions in adults is crucial for developing preventive public health strategies.

Digital addiction and movement behaviours are two interrelated domains of growing public health concern [25,29,30]. However, their relationship has not been adequately examined in adult populations. To our knowledge, no previous study has comprehensively examined the factors influencing sedentary behaviour among Turkish adults. Thus, the aim of this study was to identify the predictors of sedentary behaviour in adults, with a particular focus on the roles of social media addiction and physical activity, while controlling for relevant sociodemographic and behavioural factors. We hypothesised that sedentary behaviour would be associated with higher levels of social media addiction and lower levels of physical activity in adults. Additionally, we expected that these associations would remain significant after controlling for sociodemographic and behavioural factors.

## METHODS

### Study design and setting

This study employed a cross-sectional analytical research design. A snowball sampling strategy was used, which may limit the sample's representativeness due to potential selection bias. [31]. The Google Forms link was shared by researchers via WhatsApp. Detailed information was added as an explanation. This link included consent forms and evaluation forms. The study was conducted online with volunteer participants between April and June 2025.

Ethics committee approval number E-37077861-900-168456 was obtained from the Iğdır University Non-Interventional Clinical Research Ethics Committee on 26 March 2025 regarding the appropriateness of conducting the research. All study procedures were conducted in accordance with the ethical standards of the National Research Committee and the Declaration of Helsinki [32]. A voluntary consent form was obtained from the adults participating in the study through Google Forms.

This study was reported in accordance with the STROBE (Strengthening the Reporting of Observational Studies in Epidemiology) guidelines for observational studies.

### Participants

The study population consisted of adults aged 18 to 65 years living in Turkey. The sample size was calculated to be at least 384 participants, assuming a 95% confidence level, a 5% sampling error, and an unknown population size [33]. The criteria for inclusion in the study were knowing Turkish, being between the ages of 18 and 65, agreeing to participate, and being a smartphone and social media user. The exclusion criteria were having an acute musculoskeletal problem that would prevent physical activity and a problem that would prevent smartphone use.

### Data collection and outcome measures

The Sociodemographic Data Form developed by the researchers, the Bergen Social Media Addiction Scale, the Sedentary Behavior Scale, and the International Physical Activity Questionnaire-Short Form were used to collect the data.

#### Sociodemographic Data Form

In the form developed by the researchers, in line with the literature, the sociodemographic characteristics of the participants (age, gender, educational status, employment status, income status, body mass index, and daily internet use time) were assessed.

#### Bergen Social Media Addiction Scale

The scale was developed by Andreassen et al. to measure the levels of addiction to social media use [34]. Each item in the scale was prepared based on six basic addiction criteria: mental effort, mood alteration, tolerance, deprivation, conflict, and failed cessation attempt. The scale is a 5-point Likert type. The scale yields a minimum of six and a maximum of 30 points, with no reverse-scored items. As the average scores obtained from the scale increase, the level of social media addiction increases proportionally. Cut off 19 [35]. The internal consistency coefficient (Cronbach's Alpha) of the scale is 0.83 [36]. In this study, the Cronbach’s alpha coefficient for the Bergen Social Media Addiction Scale was 0.839.

#### Sedentary Behavior Questionnaire (SBQ)

The Sedentary Behavior Scale (SBQ) was developed by Rosenberg et al. (2010) to determine the time spent sitting during the day [37]. The scale consists of 11 items related to sedentary lifestyle, such as watching television, using a computer, listening to music, and working at a desk, both on weekdays and weekends. To indicate the time spent on these activities, the following answers can be given: ‘none’, ‘15 minutes or less’, ‘30 minutes’, ‘1 hour’, ‘2 hours’, ‘3 hours’, ‘4 hours’, ‘5 hours’, ‘6 hours or more’. The time spent on each behaviour, marked before scoring, is converted to hours (for example, an individual's 15-minute answer is recorded as 0.25 hours). The total duration of sedentary behaviour for individuals is calculated by summing hours separately on weekdays and weekends. If the duration of sedentary behaviour on an average day during or at the end of the week exceeds 24 hours, it is reduced to 24 hours and recorded. For total time, the average time on a weekday is multiplied by five, the average one-day time on the weekend is multiplied by two, and the total average weekly sedentary time is recorded [38]. The item and total score of the scale demonstrated moderate to excellent reliability for weekdays (range = 0.64–0.90) and weekend days (range = 0.51–0.93) [37]. In this study, the Cronbach’s alpha coefficient for the SBQ weekdays was 0.702 and for the SBQ weekend days was 0.709.

#### International Physical Activity Questionnaire-Short Form (IPAQ-SF)

The survey was developed in 1998 by an international group of experts under the leadership of the World Health Organization (WHO) to measure physical activity levels among individuals aged 15–69. The International Physical Activity Questionnaire-Short Form (IPAQ-SF) measures the frequency, duration, and intensity of physical activities performed over the past seven days, enabling the calculation of metabolic equivalents (MET) and the quantification of weekly physical activity. Survey data are evaluated using Metabolic Equivalent (MET) scores and calculated as weekly work hours (MET-min/week). Based on the calculated MET value, individuals' physical activity levels are classified as ‘<600 MET-min/week’ inactive, ‘600-3000 MET-min/week’ minimally active, and ‘>3000 MET min/week’ very active (physical activity considered beneficial for health). The test-retest reliability correlation coefficient was reported as r = 0.76 [39].

These instruments were selected to capture interconnected behavioural domains within a comprehensive movement behaviour framework. Examining sedentary behaviour alongside social media addiction, and considering daily internet use time as an additional behavioural indicator, enables a multidimensional assessment of how digital engagement patterns relate to overall movement behaviours in adults.

After data collection, the data set was screened for implausible values. For sedentary behaviour, daily total duration exceeding 24 hours was considered implausible and was truncated to 24 hours [38]. In the present study, six participants reported sedentary time exceeding 24 hours per day; these values were adjusted accordingly. IPAQ responses were processed in accordance with established scoring guidelines. Participants reporting more than 16 hours (960 minutes) of total physical activity per day were to be excluded as implausible. However, no cases met this exclusion criterion in the present data set.

### Data collection process

The data was collected online using a Google Form. The study was conducted with individuals who agreed to participate and completed the consent form. Participants were asked to complete the online questionnaires independently; however, those unable to do so were permitted to receive assistance. The data collection process took an average of 15–20 minutes for each participant.

### Analysis of data

The data obtained in this study were analysed using IBM SPSS Statistics for Windows, Version 23.0 (IBM Corp., Armonk, NY, USA). The data were examined for normality to determine the appropriate statistical method for analysis [40]. Pearson correlation was used for variables with a normal distribution, whilst Spearman’s rank correlation was used for variables that were not normally distributed. The magnitude of the correlation was classified as follows: ≥0.80, strong; 0.70 to 0.40, moderate; <0.40, weak [41]. Finally, a four-stage hierarchical regression analysis was conducted to identify the determinants of a sedentary behaviour.

## RESULTS

The mean age of 442 individuals was 31.65 ± 11.99 years, with 33.9% male and 66.1% female. Regarding educational level, 60.4% had an education below university, while 39.6% held a university degree or higher. Additionally, 62.2% were unemployed, and 54.5% reported a balanced income-expenditure status. The mean body mass index was 24.62 ± 4.51, and the average daily internet usage time was 3.76 ± 2.44 hours (**Table 1**).

**Table 1:** Distribution of demographic characteristics of individuals participating in the study

Characteristics	n *=* 442, %
Gender	
*Male*	150 (33.9)
*Female*	292 (66.1)
Educational level	
*<University*	267 (60.4)
*≥University*	175 (39.6)
Employment level	
*Unemployed*	275 (62.2)
*Employed*	167 (37.8)
Socioeconomic status	
*Income less than expenses*	134 (30.3)
*Income equals expenses*	241 (54.5)
*Income exceeds expenses*	67 (15.2)
Age (mean ± SD)	31.65 ± 11.994
Body mass index (mean ± SD)	24.62 ± 4.507
Daily Internet use duration (mean ± SD)	3.76 ± 2.435

SD – standard deviation

A weak positive relationship was found between social media addiction and sedentary behaviour (r = 0.294, *P* < 0.001). A negative relationship was observed between social media addiction and physical activity levels (r = −0.124, *P* = 0.009), whereas no significant correlation was found between sedentary behaviour and physical activity levels (r = 0.020, *P* = 0.679) **(****Table 2****).**

**Table 2:** Correlations between the study scales

	X ± SD		SBQ	IPAQ-SF	Social media addiction
**SBQ**	12.75 ± 5.559	r^a^	1		
		p			
**IPAQ-SF**	2623.94 ± 3680.483 (MD = 1381.50)	r^b^	−0.028	1	
		p	0.558		
**Social media addiction**	14.63 ± 5.624	r^a^	0.294	−0.124	1
		p	0.000*	0.009†	

IPAQ-SF – International Physical Activity Questionnaire-Short Form, r^a^ – Pearson correlation, r^b^ – Spearman Rank correlation, SBQ – Sedentary Behavior Questionnaire

*P < 0.01.

†*P* < 0.05.

The results of a 4-step hierarchical regression analysis to identify predictors of sedentary behaviour are presented. Demographic variables (gender, education level, income level, employment status, age, and body mass index (BMI)) were included as predictors in the first step, and the model explained 9.5% of the variance in sedentary behaviour in the total sample (F(7, 434) = 7.367, *P* < 0.001). According to the results, being male (β = −0.183, *P* < 0.001), having a high school education or lower (β = −0.139, *P* < 0.05), having an income lower than expenses (β = −0.149, *P* < 0.05), being employed (β = 0.142, *P* < 0.05), and younger age (β = −0.192, *P* < 0.001) were significant predictors of sedentary behaviour (**Table 3**).

**Table 3:** Hierarchical regression analysis results for sedentary behaviour (n = 442)

Standardised Regression Coefficients (τ) and significance (p)				
**Independent variables**	**Model 1**	**Model 2**	**Model 3**	**Model 4**
Gender				
*Male^a^*				
*Female (β, p)*	−0.183, 0.000*	−0.193, 0.000*	−0.158, 0.000*	−0.16, 0.000*
Education level				
*<University^a^*				
*≥University (β, p)*	−0.139, 0.030†	−0.149, 0.020†	−0.145, 0.015†	−0.153, 0.011†
Income level				
*Income less than expenses^a^*				
*Income equals expenses (β, p)*	−0.149, 0.005†	−0.132, 0.013†	−0.105, 0.035†	−0.099, 0.047†
*Income exceeds expenses (β, p)*	−0.044, 0.422	−0.041, 0.454	−0.025, 0.626	−0.026, 0.609
Employment level				
*Unemployed^a^*				
*Employed (β, p)*	0.142, 0.026†	0.119, 0.065	0.133, 0.028†	0.139, 0.021†
Age (β, p)	−0.192, 0.001*	−0.182, 0.001*	−0.005, 0.929	0.007, 0.904
Body mass index (β, p)	0.046, 0.349	0.039, 0.436	0.010, 0.823	0.010, 0.830
Physical activity (IPAQ-SF) (β, p)		−0.092, 0.058	−0.065, 0.155	−0.047, 0.313
Daily internet use time (β, p)			0.378, 0.000*	0.328, 0.000*
Social media addiction (β, p)				0.105, 0.044†
F	7.367	6.938	13.709	12.833
R	0.326	0.337	0.471	0.479
aR^2^	0.095	0.097	0.206	0.212
**ΔR^2^**		0.007	0.109*	0.007†

IPAQ-SF – International Physical Activity Questionnaire-Short Form, τ– standardised regression coefficient, aR^2^ – adjusted R square, ΔR^2^ – Delta R-Square (Change)

*P < 0.01.

†*P* < 0.05.

Physical activity was entered in the second step of the model. With the inclusion of physical activity, the overall model remained statistically significant (F(8, 433) = 6.938, *P* < 0.001); however, the contribution of physical activity to the model was not significant (ΔR^2^ = 0.007, *P* > 0.05). Accordingly, physical activity did not significantly predict sedentary behaviour (β = −0.092, *P* = 0.058).

Daily internet use time was entered in the third step of the model. With its inclusion, the overall model remained statistically significant (F(9, 432) = 13.709, *P* < 0.001), and the explained variance increased significantly (ΔR^2^ = 0.109, *P* < 0.001). The model accounted for 20.6% of the variance in sedentary behaviour (aR^2^ = 0.206). Daily internet use time emerged as a significant predictor of sedentary behaviour (β = 0.378, *P* < 0.001), indicating that higher internet use time was associated with higher levels of sedentary behaviour.

**Figure 1:**
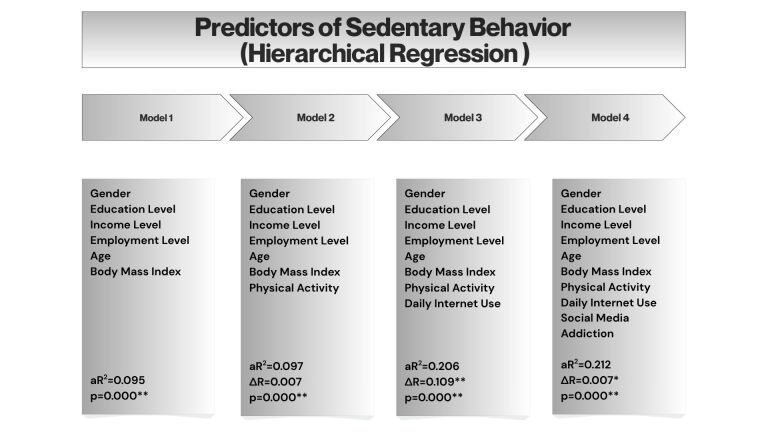
Predictors of sedentary behaviour.

Social media addiction was entered in the final step of the model. With its inclusion, the overall model remained statistically significant (F(10, 431) = 12.833, *P* < 0.001), and social media addiction contributed significantly to the model (ΔR^2^ = 0.007, *P* < 0.05). The final model explained 21.2% of the variance in sedentary behaviour (aR^2^ = 0.212). Social media addiction emerged as a significant predictor of sedentary behaviour (β = 0.105, *P* = 0.044). Multicollinearity diagnostics indicated no significant issues across all models (VIF<10). Additionally, the Durbin-Watson statistic for the final model was 1.845, suggesting no substantial autocorrelation among the residuals (**Figure 1**).

Effect size analysis (Cohen’s f^2^) indicated negligible effects for physical activity (f^2^ ≈ 0.008) and social media addiction (f^2^ ≈ 0.009), and a small-to-moderate effect for daily internet use time (f^2^ ≈ 0.14).

## DISCUSSION

This study aimed to identify the predictors of sedentary behaviour in adults, with a particular focus on the roles of social media addiction and physical activity while controlling for relevant sociodemographic and behavioural factors. The findings from the hierarchical regression analyses indicated that, while social media addiction contributed significantly to the model, its effect size was relatively small. Whereas physical activity did not emerge as a significant predictor when these factors were accounted for. Daily internet use time was the strongest predictor of sedentary behaviour. In addition, several sociodemographic variables, including age, gender, education level, income status, and employment status, were associated with sedentary behaviour. Overall, the results suggest that sedentary behaviour in adults is influenced by a combination of demographic and behavioural factors, with digital addiction showing a modest but significant contribution beyond traditional sociodemographic characteristics.

Previous research has shown that smartphone use/addiction is associated with higher sedentary behaviour in adolescents and young adults [42–45]. However, research on social media addiction in adults is quite limited in the literature. Fennel et al. observed an association between mobile phone use and sedentary behaviour in adults [46]. Similarly, another study reported that American adults spend considerably more time sitting than previously estimated, with a substantial portion of their leisure time spent on internet-based activities [47]. In the present study, sedentary behaviour was more strongly associated with daily time spent on the internet than with social media addiction. Given the relatively small effect sizes observed, these findings should be interpreted with caution and considered as evidence of modest associations rather than strong behavioural determinants. During the COVID-19 pandemic, increased screen time was accompanied by a rise in sedentary behaviour [30]. However, sedentary behaviour related to screen time may not necessarily correspond to more complex constructs such as addiction. This may be because addiction involves additional dimensions, including compulsive use and withdrawal symptoms, which may not be fully captured by general patterns of sedentary behaviour.

Consistent with the findings of Bauman et al. [48], younger age emerged as a significant predictor of sedentary behaviour, indicating that younger individuals tended to have higher levels of sedentary behaviour. Previous research has also reported higher levels of sedentary behaviour among young adults and has attributed increased sitting time to greater engagement in digital activities [49,50]. In addition, our results indicate that younger individuals exhibited higher levels of sedentary behaviour alongside higher physical activity and social media addiction levels. This pattern may reflect a tendency toward the ‘active couch potato’ [4] phenomenon among younger adults. Some studies have reported that sedentary behaviour increases with age [46,51,52]. These inconsistencies may reflect differences in sample composition, particularly the inclusion of very old adults (*e.g.*≥80 years) in some studies, whereas our study included participants aged 18–65 years. Variations in measurement methods and contextual factors may also contribute to these divergent findings. Given the well-established associations between prolonged sedentary behaviour and adverse health outcomes, the higher levels of sedentary behaviour observed among younger adults are noteworthy. However, longitudinal research is needed to better understand age-related trajectories.

Higher educational attainment is generally associated with an increased likelihood of engaging in desk-based occupations, which may lead to higher levels of sedentary behaviour [49,53,54]. It has also been suggested that higher socioeconomic status, often linked to education, may contribute to increased sedentary behaviour [55,56]. However, an interesting finding was that having an income lower than expenses was associated with higher sedentary behaviour. This may be related to limited access to leisure-time activities due to lower socioeconomic status, which could result in increased sedentary behaviour. Although higher BMI has been theoretically associated with sedentary behaviour, the evidence in the literature remains inconclusive [55]. Similarly, in the present study, sedentary behaviour was not associated with BMI.

In the present study, male gender was associated with higher levels of sedentary behaviour. The relationship between sedentary behaviour and gender is inconsistent in the literature [57]. While some studies report no association [46,58], others indicate that sedentary behaviour varies according to gender [51,49,59]. These differences may be attributable to the association of gender-related findings with the cultural characteristics of the study sample, the study design, and the assessment tools used. Furthermore, as both our study and much of the existing literature rely on self-reported measures, potential recall errors, social desirability bias, and misclassification may have influenced the observed gender-related patterns. In particular, the physical activity questionnaire may have been challenging for some participants to interpret accurately, potentially leading to reporting errors or misclassification of activity levels. Additionally, when assessing sedentary behaviour, certain daily habits may conceptually overlap across subcategories, and some behaviours could have been reported under more than one heading. Such overlap may have influenced the estimation of sedentary time. Indeed, it is well established that self-reported measures tend to yield higher estimates of sedentary time compared to device-based assessments [60,61]. Therefore, the findings should be interpreted with caution, and future studies using objective measurement tools are warranted to provide more precise estimates.

The lack of objective methods to measure sedentary behaviour, physical activity, and social media addiction is a significant limitation of our study [51,62]. All variables were assessed using self-report instruments, which may be subject to recall bias and social desirability bias. Participants may have underreported sedentary time or overreported physical activity levels, potentially influencing the observed associations. However, objective methods may make it difficult to reach the desired number of participants, as they require contacting participants individually. The use of snowball sampling may have introduced selection bias and limited generalisability. Certain subgroups – particularly males and individuals with limited digital access, lower socioeconomic status, or older age – may have been underrepresented.

Despite being conducted in Turkey, the study's findings may be applicable to other sociocultural and economic contexts, particularly those of low- and middle-income countries undergoing rapid digitalisation and rising sedentary behaviour. It is imperative to exercise caution when drawing generalisations from observational studies. Further research is necessary to validate these patterns in diverse settings and to ensure the generalisability of the findings.

## CONCLUSIONS

Beyond statistical significance, the magnitude of the observed effects indicates that although digital use is associated with sedentary behaviour, its impact varies depending on how it is operationalised. General usage patterns, such as daily internet use duration, appear to be more influential than addiction-related constructs. Notably, sedentary behaviour was not significantly associated with physical activity, highlighting the importance of considering these behaviours as distinct constructs. Given the well-established links between sedentary behaviour and adverse health outcomes, the independent evaluation of sedentary behaviour should not be overlooked. These findings may serve as a basis for informing public health strategies; however, intervention studies are needed before translating these results into concrete public health recommendations.
